# Metastasis in biological time

**DOI:** 10.3389/fcell.2026.1845678

**Published:** 2026-06-25

**Authors:** Jhommara Bautista, Sofía Ojeda-Mosquera, Camila Beltrán-Flores, David Palacios-Zavala, Andrés López-Cortés

**Affiliations:** Cancer Research Group (CRG), Faculty of Medicine, Universidad de Las Américas, Quito, Ecuador

**Keywords:** biological time, circadian immune dynamics, circadian system, metastasis, precision oncology, stromal rhythms, tumour-intrinsic clocks, zeitgebers

## Abstract

Metastasis is the leading cause of cancer-related death, yet it is still interpreted largely through genetic, spatial, and microenvironmental models that treat the host as temporally uniform. Emerging evidence challenges this view by showing that metastatic progression unfolds within a hierarchically organized circadian system in which tumour cells, vascular interfaces, immune compartments, and distant organs operate in biological time. In this review, we examine how circadian regulation shapes multiple stages of the metastatic cascade, including tumour metastatic competence, circulating tumour cell release, vascular trafficking, immune-mediated seeding, niche permissiveness, colonization, and metastatic outgrowth. We discuss how tumour-intrinsic clocks influence invasive and secretory programmes, how endothelial and stromal rhythms create time-dependent windows of tissue access, and how circadian immune dynamics determine whether disseminated tumour cells are eliminated or licensed for persistence. We further highlight the role of systemic zeitgebers, including light–dark cycles, feeding–fasting rhythms, glucocorticoids, autonomic signalling, and body temperature, in coordinating or disrupting temporal alignment across metastatic compartments. Together, these observations support a conceptual shift: metastasis should be understood not only as a disease of space and state, but also as a disease of biological time. This framework may help explain metastatic heterogeneity and inform future precision oncology strategies.

## Introduction

Metastasis is the leading cause of cancer-related death, yet it is still interpreted largely through spatial and genetic frameworks that explain where tumour cells spread and which molecular programmes they acquire, but not when the decisive steps of metastatic progression occur (Gerstberger et al., 2023; Gupta and Massagué, 2006). Dissemination, vascular arrest, immune escape and colonization are often analysed as if they unfolded in a physiologically uniform host. In reality, these events occur within an organism whose vascular, endocrine, immune and metabolic states are rhythmically coordinated across the day. Metastasis, therefore, is not only a disease of space and state, but also a disease of biological time (Koronowski and Sassone-Corsi, 2021; Wang et al., 2022).

In mammals, circadian timing is organized hierarchically (Takahashi, 2017). The suprachiasmatic nucleus (SCN) aligns behaviour and systemic physiology with the light–dark cycle. Peripheral clocks in nearly all tissues then impose local temporal control over transcriptional programmes, metabolism, trafficking, stress responses, and endocrine sensitivity (Bautista et al., 2025c; Lee, 2021; Bautista et al., 2025b). These oscillators do not merely keep time; they determine when tissues are receptive to nutrients, responsive to hormones, permissive to cellular entry, and able to mount inflammatory, reparative, or suppressive programmes (Koronowski and Sassone-Corsi, 2021; Wang et al., 2022). Because tumour cells disseminate, circulate, and seed within a temporally structured host, each stage of the metastatic cascade unfolds within rhythmic physiology. The probability that a tumour cell enters the bloodstream, survives transit, gains access to a distant organ, or progresses from silent persistence to overt outgrowth may therefore depend not only on tumour-intrinsic properties, but also on the circadian state of the host (Diamantopoulou et al., 2022; Casanova-Acebes et al., 2018; Wang et al., 2024).

This possibility is now supported by convergent evidence. Tumour cells can retain or rewire circadian programmes in ways that influence epithelial–mesenchymal plasticity, invasion and secretory behaviour (Li et al., 2024; Jung et al., 2013). Circulating tumour-cell (CTC) release is temporally patterned rather than continuous, and the metastatic properties of CTCs vary according to the time of release. In parallel, endothelial and stromal clocks shape vascular trafficking and tissue access, whereas rhythmic immune surveillance influences whether disseminated cells are eliminated or allowed to persist (Diamantopoulou et al., 2022; Wang et al., 2024; Casanova-Acebes et al., 2018). Systemic zeitgebers, including feeding–fasting cycles, glucocorticoid rhythms, sympathetic tone, body temperature, melatonin, and sleep–activity patterns, can synchronize or desynchronize peripheral clocks across tumour and host tissues, thereby reshaping the temporal conditions under which metastasis progresses. Circadian biology, therefore, acts not only at isolated steps of the metastatic cascade, but as a systems-level regulator of metastatic progression (Koronowski and Sassone-Corsi, 2021; Oster et al., 2017).

**FIGURE 1:**
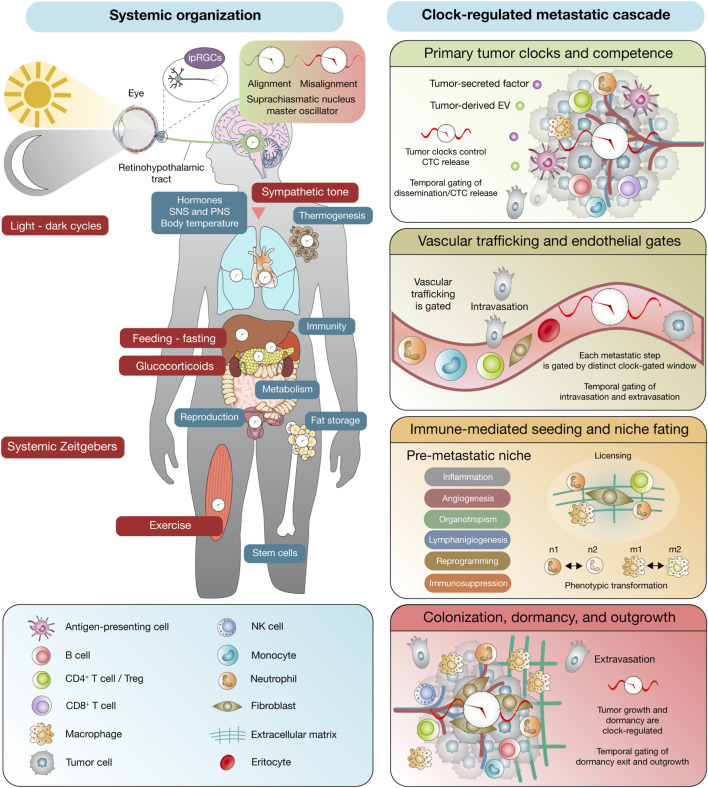
Circadian system organization and clock-regulated control of the metastatic cascade. The left panel illustrates the hierarchical systemic organization of circadian timing. Light–dark cycles entrain the suprachiasmatic nucleus (SCN) through intrinsically photosensitive retinal ganglion cells and the retinohypothalamic tract, coordinating central alignment or misalignment states. The SCN distributes temporal information to peripheral tissues through systemic zeitgebers, including autonomic tone, hormones, body temperature, feeding–fasting cycles, glucocorticoids, and exercise, thereby regulating tissue-level functions relevant to metastasis, including immunity, metabolism, thermogenesis, fat storage, reproduction, and stem-cell biology. The right panel summarizes how circadian regulation may shape successive stages of the metastatic cascade. Primary tumour clocks influence metastatic competence, tumour-secreted factors, extracellular vesicle release, and the temporal gating of circulating tumour-cell (CTC) dissemination. Host vascular and endothelial clocks regulate intravasation, trafficking, and extravasation through time-dependent adhesive and barrier states. Immune-mediated seeding and pre-metastatic niche formation are likewise temporally regulated through inflammatory, angiogenic, lymphangiogenic, stromal, and immunosuppressive programmes. Finally, colonization, dormancy, and metastatic outgrowth are represented as clock-regulated processes within the distant organ microenvironment. Together, the figure presents metastasis as a biologically time-structured process emerging from the interaction between tumour-intrinsic clocks, host systemic organization, and peripheral tissue permissiveness.

Yet the role of biological time in metastasis remains conceptually fragmented. Circadian research in cancer has often emphasized clock-gene dysregulation, tumour proliferation and chronotherapy, whereas metastasis research has largely focused on clonal evolution, niche biology and immune escape without treating temporal regulation as a core organizing principle (Karaboué et al., 2024; Gerstberger et al., 2023). Here, we synthesize these domains by proposing that metastasis should be understood as a temporally regulated, multistage process unfolding within a distributed circadian system (Figure 1). Rather than treating clocks as secondary modifiers of tumour behaviour, we frame circadian organization as a biologically consequential layer that shapes metastatic competence, dissemination, vascular trafficking, immune surveillance, colonization, and clinical response. This perspective does not replace tumour-centric or microenvironmental models of metastasis; it extends them by placing metastatic progression in biological time. Importantly, this framework does not imply that metastatic timing is identical across all cancers. Instead, it proposes that biological time is a context-dependent regulatory layer whose effects may vary according to tumour type, metastatic route, target organ, host physiology, and tumour–host circadian alignment.

## The systemic architecture of metastatic timing

Metastasis does not unfold in a temporally neutral host. It arises within a hierarchically organized circadian network in which the SCN functions as the central pacemaker while peripheral clocks in tissues relevant to metastasis, including liver, lung, bone marrow, endothelium, and immune compartments, generate local oscillations that are synchronized, but not fully dictated, by central time (Damiola et al., 2000; Yoo et al., 2004; Méndez-Ferrer et al., 2008). This distinction is fundamental. The host is not governed by a single homogeneous clock, but by a distributed network of oscillators whose compartments can remain aligned, drift apart transiently or become chronically desynchronized. As a result, dissemination, vascular access, immune surveillance, metabolic support, and target-organ receptivity do not occur in a fixed physiological background, but within a shifting temporal landscape (Scheiermann et al., 2012; Brown et al., 2002).

This distributed architecture is not simply theoretical. Restricted-feeding studies in nocturnal mice showed that daytime feeding shifts peripheral clock-gene expression in the liver by up to 8–12 h, whereas Per1 and Per2 rhythms in the SCN remain largely unchanged (Damiola et al., 2000; Stokkan et al., 2001). Importantly, this uncoupling persisted in constant darkness, demonstrating that feeding can reset peripheral clocks independently of the light-entrained central pacemaker. Re-entrainment was gradual rather than abrupt, and different peripheral tissues reset at distinct rates, indicating that they can occupy different circadian phases during adaptation. These experiments establish that compartments relevant to metastasis can become temporally misaligned from the SCN and from one another (Damiola et al., 2000; Stokkan et al., 2001). Because the term zeitgeber is central to this framework, we provide a dedicated explanation of its meaning and relevance to metastatic timing in Box 1.

Body temperature provides a second mechanistically powerful example. Temperature cycles within the physiological range were sufficient to induce or sustain rhythmic circadian gene expression in fibroblasts, and natural body-temperature rhythms maintained previously induced oscillations that would otherwise dampen at constant temperature (Brown et al., 2002). *In vivo*, altered ambient temperature cycles rephased peripheral tissues without equivalently resetting the SCN. Buhr and colleagues sharpened this principle further using Per2Luc tissues *ex vivo*, showing that peripheral organs were strongly reset by modest temperature pulses, whereas the adult SCN remained resistant unless neuronal coupling was disrupted. Together, these experiments demonstrate that a host-derived systemic cue can impose timing on peripheral tissues while leaving the central pacemaker relatively stable (Brown et al., 2002; Buhr et al., 2010). For metastasis, this implies that a single endocrine or physiological cue may realign some tissues while leaving others anchored to central time, thereby generating internal phase divergence across metastatic compartments (Damiola et al., 2000).

Three concepts are therefore central. Circadian disruption refers to the breakdown of normal rhythmic organization induced by environmental or behavioural perturbations such as chronic jet lag, shift work, light-at-night exposure, and sleep fragmentation (Potter et al., 2016; Galinde et al., 2022). Clock rewiring refers to altered phase, amplitude, or transcriptional architecture within tumour or stromal cells despite partial retention of rhythmicity (Li et al., 2024). Zeitgeber misalignment refers to a state in which synchronizing signals such as feeding time, body temperature, glucocorticoids, autonomic input, or metabolic cues reset some peripheral clocks more strongly than others, producing internal phase divergence despite continued SCN rhythmicity (Balsalobre et al., 2000; Brown et al., 2002; Bautista et al., 2025c). Metastasis is unlikely to be driven by clock disruption in a generic sense. Rather, the biologically relevant condition may often be systemic circadian misalignment, in which tumour, bone marrow, vasculature, immune system, and distant organs no longer share a common temporal program.

Experimental cancer models support the importance of this framework. In a spontaneous mammary tumour model, chronic jet lag disrupted systemic circadian organization and increased tumour-cell dissemination and lung metastasis, indicating that host-level temporal instability can promote metastatic progression (Hadadi et al., 2020). The central implication is not simply that circadian disruption worsens cancer, but that destabilizing systemic rhythmic organization is sufficient to make the host more permissive to metastatic progression. Metastatic efficiency may therefore depend not only on what tumour cells are capable of doing, but also on whether the host’s circadian network remains coherent enough to constrain them. Under aligned conditions, host compartments may present temporally coordinated barriers to dissemination; under misaligned conditions, tumour, endothelium, immune system, and target organs may occupy discordant phases, generating asynchronous windows of permissiveness (Hadadi et al., 2020; Scheiermann et al., 2012; Damiola et al., 2000). This systems-level temporal architecture forms the conceptual foundation for the entire review.

## Tumour clocks and metastatic competence

Before tumour cells disseminate, they must first acquire a metastatically competent state. This transition emerges within the primary tumour, where malignant cells progressively gain plasticity, invasive capacity, and stress-adaptive traits required to detach from the primary lesion and initiate dissemination (Pastushenko et al., 2018; Hadadi et al., 2020; Jung et al., 2013; López-Cortés et al., 2022). The tumour circadian clock should therefore not be understood as a passive marker of timekeeping, but as an active regulator of metastatic readiness. A key point is that circadian status in cancer is not a simple binary of intact versus lost. Tumour cells may retain, dampen, fragment, or reprogramme oscillatory circuitry, and these distinct configurations can shape metastatic traits in different ways. Human breast cancer data support the clinical relevance of this concept, as reduced expression and weakened co-expression of core circadian clock genes have been associated with tumour progression and shorter metastasis-free survival in patients with node-negative breast cancer (Cadenas et al., 2014). The strongest tumour-intrinsic evidence indicates that the clock influences metastatic competence through three linked mechanisms: organization of epithelial–mesenchymal transition (EMT)-linked transcriptional plasticity, control of invasion signalling, and regulation of prometastatic secretory programmes (Li et al., 2024; Jung et al., 2013; Dong et al., 2022).

The clearest evidence that preserved tumour rhythmicity can support metastatic behaviour comes from luminal A breast cancer. Time-stamped human breast samples, integrated with The Cancer Genome Atlas (TCGA) and the Genotype-Tissue Expression (GTEx) data through CYCLOPS 2.0, showed that luminal A tumours retain a dampened but persistent circadian organization (Li et al., 2024). Importantly, stronger rhythmic magnitude correlated with poorer 5-year survival and with increased cycling amplitude of EMT-related genes. This association was functionally validated in rhythmic MCF-7 cells, where lentiviral BMAL1 knockdown abolished reporter rhythms and significantly reduced invasion in hanging-drop and three-dimensional collagen assays. The same phenotype was reproduced in primary luminal A tumour cells and by pharmacologic clock perturbation with KL001. Notably, BMAL1 knockdown increased Ki-67 and proliferation, indicating that loss of circadian organization did not simply reduce tumour-cell viability or activity, but selectively impaired invasion while enhancing growth (Li et al., 2024). This suggests that, at least in some tumour contexts, preserved rhythmicity is co-opted to organize invasion-related states rather than suppress malignant behaviour.

A second tumour-intrinsic mechanism is the direct regulation of invasion circuitry. Jung et al. addressed this experimentally in lung cancer and glioma cells by manipulating BMAL1 expression through siRNA-mediated knockdown and expression vectors (Jung et al., 2013). Invasion was quantified using Matrigel-coated invasion chambers. BMAL1 knockdown increased invasiveness, whereas BMAL1 overexpression reduced it, and these effects were observed irrespective of p53 status. Mechanistically, loss of BMAL1 activated the PI3K–AKT–MMP2 axis, increasing PI3K activity, AKT phosphorylation, and MMP2 abundance. Pharmacologic inhibition of PI3K, AKT or MMP2 blocked the invasion induced by BMAL1 knockdown, and BMAL1 also attenuated the invasion-promoting effect of Bcl-w (Jung et al., 2013). These experiments establish a direct, causal role for a core clock component in controlling whether tumour cells engage a matrix-remodelling invasive programme.

A third mechanism is circadian control of secretory trafficking. In colorectal cancer, tumour-derived exosomes promoted the migration of both HCT116 cells and endothelial cells, and this effect was enhanced by BMAL1 overexpression and attenuated by BMAL1 knockdown. BMAL1 also increased exosome release. Mechanistically, luciferase reporter assays showed that BMAL1/CLOCK activate Rab27a transcription, directly linking the molecular clock to a core regulator of exosome secretion (Dong et al., 2022). This is important because metastatic competence is not determined only by intracellular invasion machinery, but also by the ability of tumour cells to externalize prometastatic signals through secretory pathways.

Together, these studies support a coherent view of tumour clocks as regulators of metastatic readiness. In luminal A breast cancer, retained but rewired rhythmicity supports EMT-linked cycling and invasion. In lung cancer and glioma, BMAL1 regulates invasive competence through the PI3K–AKT–MMP2 axis. In colorectal cancer, BMAL1 promotes a prometastatic secretory phenotype via Rab27a-dependent exosome release. These apparently divergent findings should not be interpreted as contradictory or as evidence that BMAL1 is uniformly pro- or anti-metastatic. Rather, they indicate that BMAL1 functions as a context-dependent regulator whose effects vary according to tumour lineage, residual clock integrity, oncogenic wiring, and the metastatic phenotype being measured. Thus, BMAL1 loss can enhance invasion in lung cancer and glioma models, whereas BMAL1 activity can support invasion-associated rhythmicity in luminal A breast cancer and promote Rab27a-dependent exosome secretion in colorectal cancer (Dong et al., 2022; Li et al., 2024; Jung et al., 2013). Their effect depends on tumour context and on the malignant networks into which they are integrated. The most defensible conclusion is that tumour circadian machinery acts as a regulator of metastatic readiness: it organizes the temporal and transcriptional conditions under which tumour cells acquire plasticity, invasion capacity, secretory adaptation, and stress-tolerant phenotypes compatible with dissemination.

### Temporal gating of tumour-cell dissemination

The transition from metastatic competence to active dissemination is not temporally uniform. Once tumour cells have acquired the traits required to leave the primary lesion, their entry into the bloodstream does not occur continuously or at random. Instead, intravasation is temporally gated, and CTC abundance follows daily oscillatory patterns (Diamantopoulou et al., 2022; Zhu et al., 2021).

The strongest direct evidence comes from breast cancer. In paired blood samples collected at 10:00 and 04:00 on the same day, most CTCs were detected during the rest phase rather than the active phase, including single CTCs, CTC clusters, and CTC–white blood cell clusters (Diamantopoulou et al., 2022). These human observations were reinforced in several mouse breast cancer models, where CTC counts also oscillated across the 24-h cycle and were enriched during the rest phase. Importantly, the same oscillatory pattern became even more pronounced when blood was sampled directly from tumour-draining vessels, indicating that the rhythm reflected differences in tumour-cell entry into the circulation rather than mere fluctuations in peripheral detection. This distinction is biologically crucial. Because CTC circulation half-life is short and clearance rates were similar between phases, the most parsimonious interpretation is that the oscillations are driven primarily by changes in intravasation rate. Tumour cells are therefore preferentially exported during specific temporal windows rather than entering the bloodstream at a constant frequency. Intravasation thus emerges as a circadian-regulated step of the metastatic cascade rather than a passive consequence of tumour pressure or vascular leakage (Diamantopoulou et al., 2022; 2023).

A complementary perspective came from an orthotopic prostate cancer model analysed by continuous fluorescence *in vivo* flow cytometry. There, CTCs were not homogeneously distributed even over short intervals, but instead appeared in stochastic bursts, particularly early in disease progression. Across 24 h, CTC counts showed marked daily oscillation, peaking at the onset of the active phase. This pattern persisted in constant darkness and could be reset by altered light–dark schedules, indicating endogenous circadian control. These experiments suggest that dissemination operates on at least two timescales: a circadian rhythm across the day and superimposed burst-like dynamics on shorter intervals (Zhu et al., 2021).

CTCs released at different times are not only quantitatively different but also qualitatively distinct. In breast cancer, rest-phase CTCs displayed greater metastatic proclivity than active-phase CTCs in functional assays. Single-cell transcriptomics showed that rest-phase CTCs were enriched for mitotic and cell-cycle programmes, whereas active-phase CTCs exhibited a different transcriptional profile. Similar oscillations in proliferation were observed within the primary tumour itself, indicating that the temporal biology of CTC release reflects a broader rhythmic organization of tumour-cell state. Thus, the bloodstream is seeded with time-stamped tumour-cell populations whose biological properties depend on when they are released (Gu et al., 2024; Diamantopoulou et al., 2023; Thomas et al., 2022).

Host endocrine rhythms contribute directly to this timed release. In breast cancer-bearing mice, dexamethasone and testosterone suppressed CTC generation, whereas insulin administration during the rest phase inverted the normal pattern of CTC abundance and tumour-cell proliferation. These manipulations show that dissemination timing is not purely tumour-autonomous, but reflects a synchronized host–tumour interaction. The bloodstream should therefore not be viewed as a neutral conduit. It is the first compartment in which metastatic progression becomes overtly rhythmic, both in the abundance and the biological state of disseminating tumour cells (Liu et al., 2022; Hadadi and Acloque, 2021; Diamantopoulou et al., 2022).

## Host clocks as gatekeepers of vascular trafficking and tissue access

After tumour cells enter the circulation, metastatic trafficking depends not only on tumour-cell fitness but also on whether the recipient tissue is in a temporally permissive state. The host vasculature is therefore not a passive route, but a rhythmic gatekeeping system whose adhesive and barrier properties fluctuate across the day (Scheiermann et al., 2012; Wang et al., 2024). The mechanistic foundation for this concept comes from studies showing that recruitment of circulating cells into tissues is under circadian control. Scheiermann and colleagues used adoptive transfers, multichannel intravital microscopy, and endothelial phenotyping to show that homing of hematopoietic cells to bone marrow and skeletal muscle oscillates across the day (Scheiermann et al., 2012). Adhesion and tissue entry correlated with rhythmic expression of endothelial P-selectin, E-selectin, VCAM1, and ICAM1, and adrenergic stimulation enhanced both endothelial adhesiveness and tissue homing. Blocking VCAM1 or using selectin-deficient recipients attenuated these effects. Local denervation and disruption of β-adrenergic signalling abolished the oscillations, demonstrating that temporal gatekeeping is imposed locally within the recipient organ (Pick et al., 2019; Zeng et al., 2024).

This principle has now been extended directly into tumour-bearing tissues. In melanoma models, time-of-day differences in tumour leukocyte infiltration reflected rhythmic entry from blood rather than differences in proliferation, apoptosis, or tissue retention (Wang et al., 2024). Acute blockade of LFA1-dependent entry abolished the oscillation, and endothelial ICAM1 expression on tumour vessels was higher during the permissive phase without changes in vascular density. Endothelial-specific loss of Bmal1 eliminated these rhythms. Although these experiments tracked immune cells, they show directly that the blood–tumour interface itself adopts different adhesive states across day (Wang et al., 2024; Pick et al., 2019). There is no reason to assume that a circulating tumour cell would encounter a temporally neutral endothelial surface under these conditions.

Temporal gatekeeping is not limited to the blood endothelium. Migration into lymphatic vessels is also rhythmic and depends on endothelial CCL21 gradients and adhesion systems including LYVE1, CD99, and JAM-A. Moreover, pericytes harbor functional clocks and can impose rhythmicity on endothelial cells in co-culture, whereas BMAL1 loss impairs vascular organization in three-dimensional systems (Holtkamp et al., 2021; Mastrullo et al., 2022; Zeng et al., 2024). These findings indicate that vascular access is controlled by a multicellular host unit comprising blood endothelium, lymphatic endothelium, and perivascular stroma.

The metastasis-oriented conclusion is straightforward. Circulating tumour cells do not encounter a constant host landscape. They move through tissues whose endothelial adhesion state, chemokine display, and perivascular organization fluctuate across the day. Successful arrest and tissue access should therefore be understood not only as consequences of tumour-cell deformability or ligand repertoire, but also as outcomes of whether the recipient tissue is in a temporally permissive state. This is the essence of temporal gatekeeping (Scheiermann et al., 2012; Wang et al., 2024; Holtkamp et al., 2021; Mastrullo et al., 2022).

## Circadian rhythms of immune-mediated seeding

Once a disseminated tumour cell reaches a distant organ, its fate depends on whether the local immune system eliminates it or permits its early survival. Crucially, this surveillance is not constant across the 24-h cycle. The balance between early elimination and early establishment is shaped by a rhythmic immune landscape (Casanova-Acebes et al., 2018; Wang et al., 2024). The clearest metastasis-stage evidence comes from the lung. In experimental melanoma metastasis, the number of metastatic foci depended on the time at which tumour cells were introduced into the circulation. This temporal difference was abolished when neutrophils were depleted or when neutrophil-specific circadian machinery was disrupted. Early tumour-cell homing to the lung likewise lost its time-of-day pattern under these conditions. These experiments show that rhythmic myeloid infiltration determines whether disseminated cells are initially retained within the target organ. This cannot be reduced to a simple model of neutrophils as uniformly antimetastatic. Transcriptomic analyses indicated that neutrophils shape circadian lung programmes linked to cell migration and carcinogenesis, suggesting that they regulate organ susceptibility as much as direct tumour-cell killing. Thus, rhythmic immune surveillance acts both through cellular cytotoxicity and through the conditioning of the receiving tissue (Casanova-Acebes et al., 2018; Pick et al., 2024).

A second major principle is that circadian disruption can convert immune surveillance into immunosuppression. In colorectal cancer models, circadian rhythm disorder increased lung metastasis while expanding MDSCs and impairing CD8^+^ T cell function. Mechanistically, suppressive myeloid cells were reinforced through metabolic and epigenetic changes that stabilized PD-L1, thereby strengthening immune escape at the metastatic site (Liu et al., 2024; Fortin et al., 2024). The biologically relevant conclusion is that circadian disruption increases the likelihood that disseminated cells survive their earliest immune encounter by shifting the local balance toward suppressive myeloid dominance and weakened cytotoxic control.

Effector lymphocyte access is also rhythmic. In melanoma models, CD8^+^ T cell infiltration varied by time of day and depended on rhythmic trafficking from blood, which in turn required an intact endothelial clock. A newly arrived tumour cell therefore encounters not a constant cytotoxic barrier, but a time-dependent immune landscape whose intensity depends both on immune-cell state and on host access gates (Wang et al., 2024; Fortin et al., 2024).

The evidence base is strongest for the myeloid–CD8 axis and less direct for NK cells, macrophages, and other lymphocyte populations in this specific metastasis-stage context. Even so, the central conclusion is clear: metastatic seeding is a temporally structured immunological decision rather than a uniform bottleneck. Circadian timing helps determine whether disseminated cells are cleared immediately or allowed to persist long enough to initiate metastatic establishment (Casanova-Acebes et al., 2018; Liu et al., 2024; Pick et al., 2024).

## Systemic zeitgebers as regulators of metastatic timing

If metastasis unfolds within a circadian organism, then its timing cannot be understood by examining tumour cells or metastatic organs in isolation. It must also be understood through the systemic signals that entrain, phase-shift, or desynchronize peripheral clocks across the host. This is the biological role of zeitgebers in metastasis. They are not abstract synchronizers, but active regulators of when peripheral tissues become more or less permissive for tumour progression (Pezük et al., 2012; Bautista et al., 2025c; Cuesta et al., 2015).

A first and especially important zeitgeber is feeding–fasting timing. Restricted feeding can reset peripheral clocks independently of the SCN, thereby reorganizing the temporal landscape in which metastasis unfolds (Damiola et al., 2000). In cancer models, time-restricted feeding improved insulin homeostasis, reprogrammed tumour circadian and metabolic rhythms, and reduced tumour progression and metastatic burden. In obesity-driven postmenopausal breast cancer, the antitumour effect of time-restricted feeding was mimicked by diazoxide and abolished by insulin pumps, indicating that insulin-related mechanisms are necessary for at least part of this phenotype. Feeding time therefore acts not only as a metabolic variable, but also as a systemic timing cue that modulates tumour-promoting physiology (Das et al., 2021; Shi et al., 2023; Kalam et al., 2023).

A second major zeitgeber is glucocorticoid rhythmicity. Glucocorticoids are outputs of the circadian system and powerful inputs to peripheral clocks (Pezük et al., 2012). In metastasis-related experiments, glucocorticoid manipulations altered the timing and magnitude of CTC generation without simply shrinking the primary tumour, indicating that endocrine timing signals can actively retime dissemination-related behaviour (Diamantopoulou et al., 2022; Khadka et al., 2023). This places glucocorticoids upstream of tumour-cell state, vascular timing, and immune surveillance.

A third zeitgeber is sympathetic tone, which distributes central time to peripheral tissues and links circadian organization to malignant progression (Lee et al., 2010; Cole et al., 2015). Disruption of the central clock–SNS–peripheral clock axis promotes tumour development and weakens peripheral tumour-suppressive programmes. Together with evidence that adrenergic signalling regulates endothelial adhesion states, this places sympathetic timing upstream of both tissue permissiveness and tumour progression (Scheiermann et al., 2012).

A fourth zeitgeber is body temperature. Physiological temperature oscillations can sustain peripheral rhythms and reset peripheral tissues without equivalently moving the SCN, making temperature a plausible determinant of temporal coherence among metastatic compartments. Its significance lies less in a single metastasis-specific readout than in its capacity to synchronize or desynchronize multiple tissues simultaneously (Brown et al., 2002; Buhr et al., 2010).

Finally, melatonin and sleep–activity cycles function as coordinated signals of biological night. Rest-phase physiology has been linked to rhythmic CTC release and metastatic proclivity, and melatonin itself can suppress invasion-associated signalling and reduce metastatic burden *in vivo*. Thus, melatonin should not be viewed merely as a hormonal marker of darkness, but as an active regulator of tumour behaviour and metastatic timing (Diamantopoulou et al., 2022; Bu et al., 2020).

These observations converge on a single principle: systemic zeitgebers regulate metastasis because they determine whether peripheral clocks across tumour, immune system, vasculature, and target organs operate in phase, out of phase, or in chronically permissive misalignment. In that sense, zeitgebers are the regulatory layer linking tumour clocks, dissemination rhythms, vascular gatekeeping, and immune surveillance into a unified systems-level model of metastatic timing (Bautista et al., 2025c).

## The temporal niche: colonization and outgrowth

Arrival in a distant organ does not guarantee metastatic success. Once seeded, tumour cells face a new set of constraints that determine whether they die, persist as solitary latent cells, remain clinically silent for prolonged periods, or initiate progressive outgrowth. Colonization is therefore a distinct bottleneck that must be separated conceptually from dissemination and early seeding (Bautista and López-Cortés, 2025; Massagué and Obenauf, 2016). This organ-specific logic is consistent with emerging organotropism frameworks showing that metastatic colonization is not random, but shaped by anatomical constraints, tumour–organ molecular crosstalk, and compatibility between disseminated tumour cells and organ-specific microenvironments (Bautista and López-Cortés, 2025). This niche permissiveness is also shaped by non-circadian systemic regulators, including the microbiome, which can influence metastatic progression through immune modulation, stromal remodelling, epithelial–mesenchymal transition, angiogenesis, pre-metastatic niche conditioning, and therapy response (Bautista et al., 2025a).

A foundational study of post-extravasation fate in mouse lung showed that metastatic inefficiency lies largely at the transition from solitary persistence to initial replication rather than at the point of arrival. Many disseminated cells remain detectable after entering the lung, but only a small fraction begins proliferating efficiently. Solitary cells are often non-proliferative and non-apoptotic, consistent with dormancy, whereas established metastases contain abundant proliferating cells. These observations define with unusual clarity the phase at which circadian control would be expected to matter most: the decision between persistence and productive outgrowth (Cameron et al., 2000; Luzzi et al., 1998; Gomis and Gawrzak, 2017).

Direct evidence that host clocks influence this phase comes from models of host Bmal1 loss. In these settings, the distant niche becomes more fibrotic and more supportive of metastatic progression through activation of the PAI1–plasmin–TGFβ axis and enrichment of myofibroblastic CAF states. Metastatic burden in the lung and liver increases markedly, and co-implantation with circadian-disrupted CAFs enhances metastatic spread (Wu et al., 2023). Early experimental models suggest that these stromal alterations influence metastatic establishment at an early post-seeding stage rather than acting only on late macroscopic growth. Importantly, inhibition of TGFβR1 reverses much of this phenotype, indicating that circadian-disruption-associated niche remodelling is mechanistically actionable (Gomis and Gawrzak, 2017; Wu et al., 2023).

Circadian disruption also reshapes the metastatic organ through immunosuppressive conditioning. In lung metastasis models, disrupted rhythmic organization was associated with accumulation of suppressive myeloid cells and dysfunctional cytotoxic lymphocytes, suggesting that persistence and outgrowth are facilitated by a distal environment less capable of elimination and more supportive of lesion expansion (Liu et al., 2024; Fortin et al., 2024).

Direct proof that dormancy versus reactivation is dictated by phase matching or mismatching between the tumour clock and the clock of the recipient organ remains limited. That idea is highly plausible and conceptually powerful, but it should still be treated as an emerging principle rather than a fully resolved fact. Even so, the current evidence is sufficient to reject the notion that metastatic colonization is temporally blind. The distant organ is a temporally regulated habitat, and when the host circadian organization is lost, the balance can shift from latency or containment toward overt metastatic growth (Massagué and Obenauf, 2016; Gomis and Gawrzak, 2017; Cameron et al., 2000).

## Translating biological time to clinical oncology

The clinical relevance of metastatic timing rests on a simple principle: if dissemination, tissue access, immune surveillance, and metastatic outgrowth are temporally structured, then oncology cannot continue to treat clock time as biologically irrelevant. The translational goal is not merely to add circadian biology to current workflows, but to develop a time-aware metastatic medicine (El-Tanani et al., 2024; Innominato et al., 2010).

A first implication is that circadian disruption should be considered a clinically relevant risk context rather than background behavioural noise. Shift work, jet lag, light-at-night exposure, sleep fragmentation, and feeding misalignment are all plausible modifiers of metastatic progression because they destabilize the temporal organization of the host (Hadadi et al., 2020; Numata et al., 2021). Importantly, however, direct human evidence linking circadian disruption to metastatic progression remains limited. Current human evidence remains stronger for biological plausibility than for definitive causal quantification of metastatic risk, but it is already sufficient to justify routine consideration of temporal disruption in clinical and epidemiological study design (Kisamore et al., 2024; Fishbein et al., 2021; Clemente-Suarez et al., 2025). At present, the strongest human support for clinically relevant biological timing comes from time-of-day variation in circulating tumour-cell abundance and phenotype, retrospective treatment-time associations, chronotherapy studies, and molecular analyses of circadian-gene alterations in metastatic cohorts. A complementary line of evidence comes from the molecular architecture of metastatic disease itself. In the pan-cancer MSK–MET cohort comprising 25,775 metastatic patients, genomic alterations in ten essential circadian rhythm-related genes (PTEN, TP53, EP300, NCOR1, GSK3B, FBXW7, PPARG, EZH2, CSNK1E, and TOP2A) were associated with substantially shorter overall survival than in unaltered cases (median 31.64 versus 55.72 months; log-rank *P* < 0.001). Although such findings do not demonstrate that treatment timing is itself causal, they reinforce a key translational point: circadian dysregulation is not merely an environmental exposure or lifestyle correlate, but a clinically meaningful dimension of metastatic biology linked to prognosis (Pérez-Villa et al., 2023)**.** Nevertheless, many mechanistic conclusions regarding dissemination, vascular trafficking, immune-mediated seeding, niche permissiveness, dormancy, and metastatic outgrowth still derive primarily from *in vivo* and *in vitro* models. Future studies should therefore validate this framework prospectively using time-stamped biospecimens, standardized liquid-biopsy timing, treatment-time annotation, circadian metadata, and tumour-type-specific clinical analyses.

The most mature clinical evidence currently concerns immune checkpoint blockade and immunotherapy-containing regimens (Bautista et al., 2026a; Kyriakidis et al., 2025). Retrospective studies in advanced cancers have shown that earlier-day administration of immunotherapy is associated with improved survival compared with later-day infusion, including studies in metastatic melanoma, mixed advanced cancer cohorts, and advanced non-small cell lung cancer treated with immunochemotherapy (Huang et al., 2025; Landré et al., 2024; Qian et al., 2021). Additional studies in metastatic and advanced-disease settings have reported concordant associations between treatment timing and clinical outcomes (Catozzi et al., 2024; Zheng et al., 2024). Although these studies do not prove causality, the consistency of the clinical signal supports treatment time as a credible, low-cost, and immediately modifiable variable for prospective evaluation. More broadly, recent precision-oncology frameworks suggest that biological time may also become a design variable for temporally controllable immunotherapies, including *in vivo* CAR T-cell platforms (Bautista et al., 2026b).

Chronomodulated chemotherapy has a longer but more heterogeneous history, particularly in metastatic colorectal cancer. Clinical trials have shown that timing chemotherapy to circadian tolerability can improve response and progression-related outcomes, but they have also revealed important modifiers such as sex, indicating that chronotherapy cannot be reduced to a universal morning-versus-evening rule. The field will therefore need precision chronotherapy rather than generic schedule shifts (Lévi et al., 1997; Giacchetti et al., 2012; Innominato et al., 2020; Printezi et al., 2022).

A second critical translational domain is biomarker timing. If CTC abundance and biological state vary with time of day, then liquid biopsy is not biologically time-invariant. Sampling time should therefore be recorded and, where possible, standardized in metastatic biomarker studies. More broadly, future temporal biomarker platforms will likely need to integrate treatment timing, blood-sampling timing, rest–activity rhythms, chronotype, and rhythm robustness rather than relying on a single clock-gene readout (Zhu et al., 2021; Ma et al., 2024).

The next-generation of studies must therefore be prospective and time-aware. At minimum, they should predefine treatment windows, record behavioural and circadian metadata, standardize biomarker sampling times, and incorporate immune or tumour readouts capable of detecting mechanisms rather than only outcomes. The clinical question is no longer only what to treat, but when to sample, when to intervene, and which patients are most likely to benefit from temporal precision (Yousefzadehfard et al., 2022; Karaboué et al., 2024; Bivolarski, 2026).

## Conclusions and future perspectives

Metastasis has long been studied through spatial, genetic, and microenvironmental frameworks, yet these models have largely treated the host as temporally uniform (Lambert et al., 2017). The evidence synthesized here argues against that assumption. Metastatic progression unfolds within a distributed circadian system in which tumour cells, vascular interfaces, immune compartments, and distant organs operate in biological time. As a result, metastatic competence, dissemination, tissue access, early survival, colonization, and therapeutic response should not be viewed as temporally neutral events. They are shaped, constrained, and at times facilitated by oscillatory programmes in both the tumour and the host (Diamantopoulou et al., 2023; Wang et al., 2023). This conclusion is supported by original evidence showing that circulating tumour-cell generation and metastatic competence can vary by rest–activity phase, that leukocyte recruitment and endothelial adhesion programmes oscillate across the day, and that immune-checkpoint-inhibitor outcomes may be associated with infusion time in retrospective clinical cohorts (Diamantopoulou et al., 2022; Scheiermann et al., 2012; He et al., 2018; Qian et al., 2021).

A central conclusion of this review is that circadian biology is not an accessory layer superimposed on metastasis, but an organizing principle that helps determine when key metastatic bottlenecks are more or less permissive. Tumour-intrinsic clocks can regulate plasticity, invasion, and secretory programmes. Dissemination is not continuous but can occur in temporally structured waves. Host endothelial, lymphatic, and stromal clocks gate vascular trafficking and tissue access, whereas immune surveillance fluctuates across the day. Systemic zeitgebers can entrain or disrupt these programmes across metastatic tissues, and the distant organ itself should be viewed as a temporally regulated habitat whose states may favour persistence, dormancy, or outgrowth (Li et al., 2024; Liu et al., 2024; Bautista et al., 2025a; Bautista and López-Cortés, 2025). Importantly, this framework does not imply that metastatic timing is identical across all cancers; rather, metastatic timing should be interpreted as context-dependent because metastatic routes, target organs, immune landscapes, and stromal niches differ substantially among tumour types (Massagué and Obenauf, 2016; Lambert et al., 2017). Together, these observations support a decisive shift in perspective: metastasis should be understood not only as a disease of space and state, but also as a disease of biological time (Table 1).

**TABLE 1:** Mechanistic evidence for circadian regulation across the metastatic cascade lllll.

Metastatic stage	Circadian-regulated process	Main mechanistic layer	Representative evidence summarized in the review	Biological implication	Translational relevance
Tumour metastatic competence	Temporal control of epithelial–mesenchymal plasticity, invasion and secretory behaviour	Tumour-intrinsic clocks; clock-controlled transcriptional and signalling programmes	Tumour cells can retain or rewire circadian programmes that influence invasion-related behaviour and secretory output, indicating that metastatic competence is not temporally static	The ability to disseminate may vary according to tumour circadian state rather than reflecting a fixed malignant trait	Tumour clock status may help explain temporal heterogeneity in dissemination risk and may identify windows of heightened metastatic competence
Dissemination and CTC release	Time-of-day variation in tumour-cell shedding into the circulation	Systemic endocrine and behavioural zeitgebers; glucocorticoids; sleep–activity phase; tumour-state retiming	The review highlights that CTC release is temporally patterned rather than continuous and that glucocorticoid manipulation alters the timing and magnitude of CTC generation without simply reducing primary-tumour size. Rest-phase physiology and melatonin-associated signals are also linked to metastatic timing	Dissemination occurs in structured waves, meaning that the bloodstream is seeded under biologically distinct host conditions across the day	Liquid-biopsy studies should record and standardize sampling time; CTC burden and phenotype should not be treated as time-invariant biomarkers
Vascular trafficking and tissue access	Rhythmic endothelial permissiveness for tumour-cell arrest, adhesion and entry into distant organs	Endothelial clocks; adhesion molecules; chemokine display; sympathetic timing	Recipient tissues are described as temporally dynamic landscapes in which endothelial adhesion state, chemokine presentation and perivascular organization fluctuate across the day, creating windows of permissive or restrictive access. Adrenergic signalling is positioned upstream of endothelial adhesion states and tissue permissiveness	Metastatic arrest is determined not only by tumour-cell properties but also by whether the host vasculature is in a temporally receptive state	Time-of-day may influence metastatic seeding efficiency and potentially the delivery or efficacy of anti-metastatic interventions targeting vascular entry
Immune-mediated seeding	Rhythmic elimination versus retention of disseminated tumour cells in the target organ	Neutrophil circadian machinery; rhythmic myeloid infiltration; CD8^+^ T-cell access	In experimental melanoma metastasis, metastatic burden depends on the time of tumour-cell entry into the circulation; this temporal effect is lost after neutrophil depletion or disruption of neutrophil circadian machinery. CD8^+^ T-cell infiltration also varies with time of day and depends on rhythmic trafficking through an intact endothelial clock	Early metastatic fate is a temporally structured immunological decision rather than a uniform bottleneck	Immunological control of metastasis may depend on treatment timing and on host circadian integrity, not only on tumour immunogenicity
Circadian disruption and immune escape	Conversion of rhythmic immune surveillance into a suppressive metastatic niche	MDSC expansion; impaired CD8^+^ T-cell function; metabolic and epigenetic PD-L1 stabilization	In colorectal cancer models, circadian rhythm disorder increased lung metastasis while expanding MDSCs, weakening CD8^+^ T-cell function and stabilizing PD-L1 through metabolic and epigenetic mechanisms	Circadian disruption does not merely dampen immunity; it can actively reprogramme the metastatic site toward permissive immune escape	Circadian misalignment should be evaluated as a modifier of metastatic risk and as a source of resistance to immunological control
Colonization, dormancy and outgrowth	Temporal regulation of the post-seeding decision between persistence, dormancy and proliferative expansion	Organ clocks; stromal and metabolic state; temporal niche receptivity	The review emphasizes that metastatic inefficiency lies largely at the transition from solitary persistence to initial replication, making colonization a distinct bottleneck and a plausible point of circadian control, although direct phase-resolved evidence remains limited	The decisive question after arrival is not only whether cells seed, but whether the organ is temporally aligned with persistence or productive outgrowth	Dormancy and metastatic awakening should be investigated in phase-resolved models that compare tumour and host circadian state directly
Systemic coordination of metastatic timing	Entrainment or desynchronization of metastatic compartments across the host	SCN output; feeding–fasting cycles; glucocorticoids; sympathetic tone; body temperature; melatonin	The review frames systemic zeitgebers as the regulatory layer that links tumour clocks, dissemination rhythms, vascular gatekeeping and immune surveillance by determining whether peripheral clocks operate in phase, out of phase or in chronically permissive misalignment	Metastasis is shaped by whole-organism temporal coherence, not only by local tumour biology	Circadian disruption should be considered a systems-level variable in metastasis models and possibly in patient stratification
Clinical translation	Time dependence of biomarker interpretation and therapeutic response	Treatment timing; biomarker sampling time; behavioural and circadian metadata	The most mature clinical signal summarized in the review concerns earlier-day immune checkpoint blockade being associated with improved outcomes in retrospective studies. The review also argues that liquid biopsy, especially CTC analysis, is biologically time-sensitive and that future studies must standardize treatment windows and sampling times	Clinical oncology cannot assume that treatment effects or metastatic biomarkers are clock-blind	Supports prospective time-aware trial design, standardized CTC sampling, and precision chronotherapy rather than generic morning-versus-evening scheduling

Evidence is now strongest for rhythmic dissemination, vascular gatekeeping, and circadian regulation of immune and stromal states, but several important questions remain unresolved (Zhu et al., 2021; He et al., 2018). It is still unclear how often tumour-cell clocks and host-organ clocks are aligned or misaligned during metastatic progression, whether successful colonization requires phase matching between disseminated cells and the recipient tissue, and how circadian disruption reshapes these interactions across different tumour types and organs. Dormancy remains a particularly attractive context for investigation, but direct evidence linking circadian phase or clock misalignment to dormant-cell maintenance and reactivation is still limited (Giancotti, 2013; Agudo et al., 2024; Liang et al., 2026). This is a critical gap because metastatic dormancy and reactivation are already known to depend on tumour-intrinsic stress programmes, extracellular matrix composition, immune surveillance, angiogenic switching, and niche-derived signals, yet these processes have rarely been investigated as time-dependent events (Aguirre-Ghiso, 2007; Ghajar et al., 2013; Park and Nam, 2020). These gaps do not weaken the framework; they define the next Frontier of the field.

Several priorities should therefore guide future work. First, metastasis studies must incorporate time as an experimental variable rather than a source of uncontrolled noise. Sampling time, lighting conditions, feeding schedules, sleep–wake disruption, and timing of tumour-cell inoculation or tissue collection should be explicitly standardized and reported (Cortés-Hernández et al., 2020). This recommendation is supported by chronobiology and pharmacology studies showing that circadian rhythms influence physiology, drug metabolism, toxicity, and therapeutic response, making uncontrolled timing a plausible source of experimental and clinical variability (Levi and Schibler, 2007; Dallmann et al., 2014; Ayyar and Sukumaran, 2021). Second, there is a need for stage-resolved circadian metastasis models that distinguish dissemination from colonization, dormancy, and outgrowth rather than collapsing them into a single metastatic endpoint (Mahmoud and Ganesh, 2024). Third, future studies should directly compare the circadian state of the tumour cell, the host vascular and immune compartments, and the recipient organ, ideally at single-cell resolution and across multiple circadian phases. Fourth, the field needs more mechanistic work in clinically relevant models to determine whether circadian disruption merely accelerates permissive processes or instead creates qualitatively distinct metastatic states (Liu et al., 2024).

A particularly important future direction will be to define the principles of tumour–host temporal coupling. Metastatic progression may depend not only on intrinsic tumour aggressiveness or on host niche receptivity, but also on whether these systems are temporally synchronized (Wang et al., 2023, 2024). A disseminated cell may arrive in an organ whose vascular, immune, or metabolic state is either aligned with its requirements or temporally hostile to them (Wang et al., 2023). Testing this possibility will require experiments that manipulate tumour-cell and host-tissue circadian phase independently, together with longitudinal approaches capable of following metastatic fate over time (Wang et al., 2024).

The translational implications are equally significant. If metastatic risk and progression are partly time-dependent, then clinical oncology must move beyond clock-blind study design (Diamantopoulou et al., 2022). Future work should evaluate circadian disruption as a modifier of metastatic risk, standardize timing in liquid-biopsy studies, and prospectively test treatment-time effects in advanced disease (Liu et al., 2024). This is particularly relevant because circulating tumour-cell abundance and phenotype can vary by sampling time, while retrospective clinical studies suggest that immune-checkpoint-inhibitor infusion time may be associated with survival outcomes in advanced cancer cohorts (Landré et al., 2024; Qian et al., 2021; Diamantopoulou et al., 2022). The goal is not to add chronotherapy superficially to existing regimens, but to build a time-aware metastatic medicine in which biological timing informs risk stratification, biomarker interpretation, and treatment scheduling (Karaboué et al., 2024).

Ultimately, the most important conceptual advance may be this: metastatic progression is not simply something that happens in the body; it happens in time. Recognizing that principle opens a new framework for understanding why metastatic success is probabilistic, heterogeneous, and context-dependent even among apparently similar tumours. The available evidence supports treating circadian organization as a testable biological variable in metastasis research, while also recognizing that temporal regulation is likely to differ across tumour types, metastatic organs, host immune states, and treatment contexts (Diamantopoulou et al., 2022; Lambert et al., 2017; Massagué and Obenauf, 2016). It also offers a path toward a more integrated chronobiology of cancer progression that is both mechanistically fertile and clinically actionable. Defining metastasis in biological time will not replace existing models of clonal evolution, niche biology, or immune escape, but it may unify them within a broader framework capable of reshaping how metastatic disease is studied—and, eventually, how it is treated.

BOX 1 **What is a zeitgeber?.**

A zeitgeber or “time giver” is an environmental or physiological cue that synchronizes circadian clocks. Light is the dominant zeitgeber for the suprachiasmatic nucleus, whereas feeding–fasting cycles, glucocorticoids, sympathetic tone, body temperature, melatonin, physical activity, and sleep–wake patterns help entrain peripheral clocks. In metastasis, zeitgebers are important because they coordinate the timing of tumour, vascular, immune, stromal, and distant-organ compartments. When these cues are aligned, host barriers to dissemination and colonization may remain temporally coordinated. When they are misaligned, different metastatic compartments may drift into different circadian phases, creating asynchronous windows of vascular access, immune suppression, niche permissiveness, or metastatic outgrowth. Thus, zeitgebers provide the mechanistic link between environmental time, systemic circadian organization, and the temporal control of metastatic progression.
